# Structure-property relationship of a model network containing solvent

**DOI:** 10.1080/14686996.2019.1618685

**Published:** 2019-06-11

**Authors:** Takeshi Fujiyabu, Yuki Yoshikawa, Ung-il Chung, Takamasa Sakai

**Affiliations:** Department of Bioengineering, Graduate School of Engineering, The University of Tokyo, Tokyo, Japan

**Keywords:** Polymer gel, model network, tetra-PEG gel, hydrogel, ideal polymer network, structure-property relationship, 10 Engineering and Structural materials, 600 Others: Polymer Gels

## Abstract

For the application of polymer gels, it is necessary to control independently and precisely their various physical properties. However, the heterogeneity of polymer gels hinders the precise control over the structure, as well as the verification of theories. To understand the structure-property relationship of polymer gels, many researchers have tried to develop a homogeneous model network. Most of the model networks were made from polymer melts that did not have a solvent and had many entanglements in the structure. Because the contribution of entanglements is much larger than that of chemical crosslinking, it was difficult to focus on the crosslinking structure, which is the structure considered in conventional theories. To overcome such a situation, we have developed a new model network system that contains much solvent. Specifically, we fabricated the polymer gel (Tetra-PEG gel) by mixing two types of solutions of tetra-armed poly(ethylene glycol) (Tetra-PEG) with mutually reactive end groups (amine (-PA) and activated ester (-HS)). Because the existence of a solvent strongly reduces the effect of entanglements, the effect of the crosslinking structure on the physical properties can be extracted. In this review, we present the structure-property relationship of Tetra-PEG gel. First, we show the structural homogeneity of Tetra-PEG gels. Then, we explain gelation reaction, elastic modulus, fracture energy and kinetics of swelling and shrinking of Tetra-PEG gels by comparing the theories and experimental results.

## Graphical abstract

**Figure:**
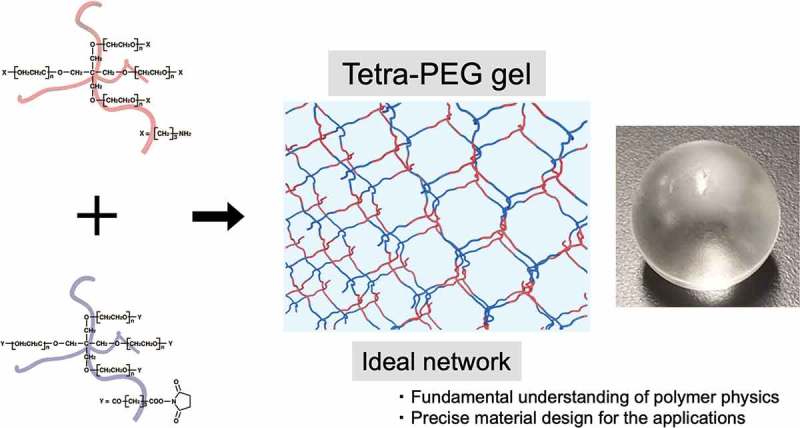

## 1. Introduction

Polymer gels consist of three-dimensional polymer networks swollen in a solvent. The application field is expanding, such as biomaterials, biosensors, drug delivery systems, templates for fabrication of nanostructures, soil modifiers, and adjuncts to shale gas extraction [1–6]. For the application of polymer gels, it is required to independently control their various physical properties: gelation, mechanical properties, swelling, degradation, and so on. However, it is generally difficult to control the physical properties of polymer gels because of the heterogeneous nature of the polymer network [7–9]. Because the heterogeneity hinders the experimental verification of the theories, it is impossible to understand the polymer gels at the molecular level. To solve this problem, many researchers attempt to develop a homogeneous model network [10–14].

Hild defined the ideal network structure as the network that satisfies the following four requirements: (i) ﻿the lengths of all network strands should be identical, (ii) ﻿the conformation of network strand should obey the Gaussian statistics, (iii) the network should be homogeneous macroscopically as well as microscopically, and (iv) the functionality of crosslinking points should be constant throughout the entire network [10]. Deviations from this definition are regarded as heterogeneities: heterogeneous distribution in strand length, density of network strands, and functionality, and abnormal statistics of network strands. Notably, this definition only focuses on the network structure characterized by chemical crosslinks, and there is no description on the other feature of the polymer network, i.e., trapped entanglement between network strands. Owing to their structure, network strands are entangled with each other to form trapped entanglements. Because the trapped entanglements are introduced in an uncontrolled manner and are difficult to be quantified, the trapped entanglements should be regarded as the heterogeneity. Thus, we propose an additional requirement for an ideal network: (v) the network strands do not entangle with each other.

A representative form of model networks is formed from a monodispersed liner prepolymer with functional groups on both ends and a multi-functional crosslinker [10,12]. The functional groups of prepolymer react with those of crosslinker to form a network structure. Due to the monodispersity of the prepolymer and defined functionality of the crosslinker, length of network strands has a narrow distribution and functionality of crosslinks is well controlled, respectively. In addition, the structure of polymers generally obeys the Gaussian distribution in a polymer melt [13,15]. Thus, this form can satisfy the requirements (i), (ii), and (iii) for an ideal network. When the two components are mixed homogeneously, the requirement (iv) may be satisfied. However, this method hardly satisfies the requirement (v), because the prepolymers strongly entangle in such a condensed condition [13,15]. Because the effect of trapped entanglements on physical properties is comparable to or surpassing that of chemical crosslinks, it is difficult to focus on that of chemical crosslinks. Especially, as for mechanical properties, the basic molecular theories describe the network structure consisting only of chemical crosslinks; therefore, the existence of a vast amount of entanglements strongly hinders the experimental verification of such theories [16]. Thus, it is important to suppress the entanglements for the fundamental understanding of polymer networks.

Dilution is the simplest method to suppress the entanglements, and a polymer network containing diluent is called a polymer gel [15,17,18]. A drawback of the coexistence of diluent is the breakage of the ideal behavior of polymer chain, i.e., the requirement (ii). Another is the intensive formation of intramolecular connections, because polymers cannot fill the space in the dilute system. Intramolecular connections lead to mechanically ineffective structures (the requirements (i) and (iv)), which are difficult to be quantified experimentally similar to the trapped entanglements [9]. The breakage of the requirement (ii) can be dissolved by using ϴ-solvent for the network strand. Thus, a molecular design to reduce the connectivity defects is a key to developing a model polymer gel.

**Figure 1.**
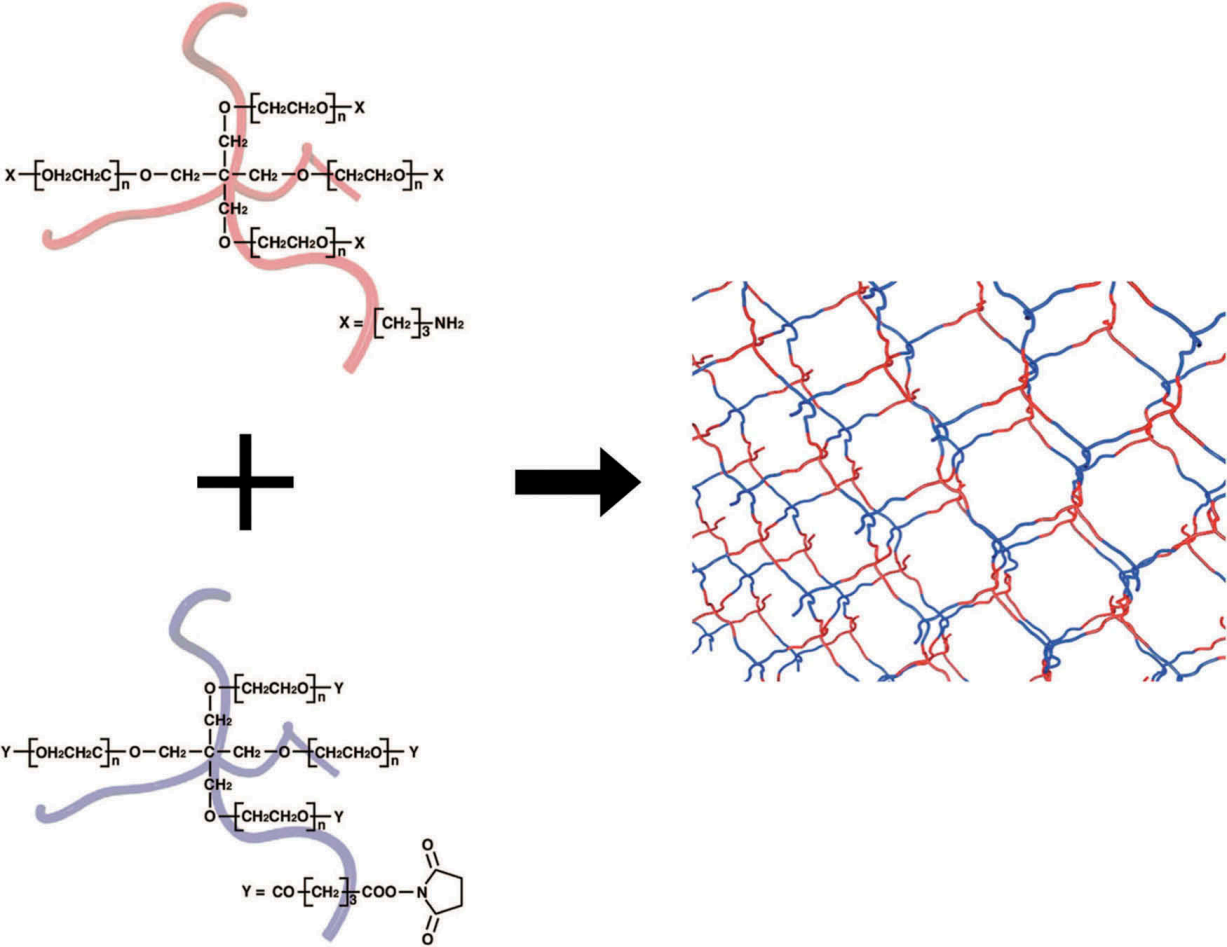
Schematic illustration of Tetra-PEG gel.

Recently, we have developed a molecular design effectively reducing the connectivity defects as well as heterogeneity [7]. The polymer gel is formed from two types tetra-armed poly(ethylene glycol) (Tetra-PEG) with mutually reactive end groups (Figure 1). This molecular design includes three keys to suppress the heterogeneity. First, the polymer concentration is set near the overlapping concentration of Tetra-PEG to suppress the entanglements. Second, the self-biting loop, which does not contribute to the elasticity, cannot be formed. Third, the reaction rate is tuned to allow the mutually reactive Tetra-PEGs to be mixed homogeneously. This molecular design realized the high reaction conversion (≈ 90%) and extremely suppressed the heterogeneity compared to conventional hydrogels [7,8,19–21]. Thus, Tetra-PEG gel is one of the promising candidates for a model network system. In this review, we present the structure of Tetra-PEG gel, and discuss physical properties: gelation reaction, elastic modulus, fracture energy and kinetics of swelling and shrinking with comparing the theoretical predictions.

## 2. Structural homogeneity

**Figure 2.**
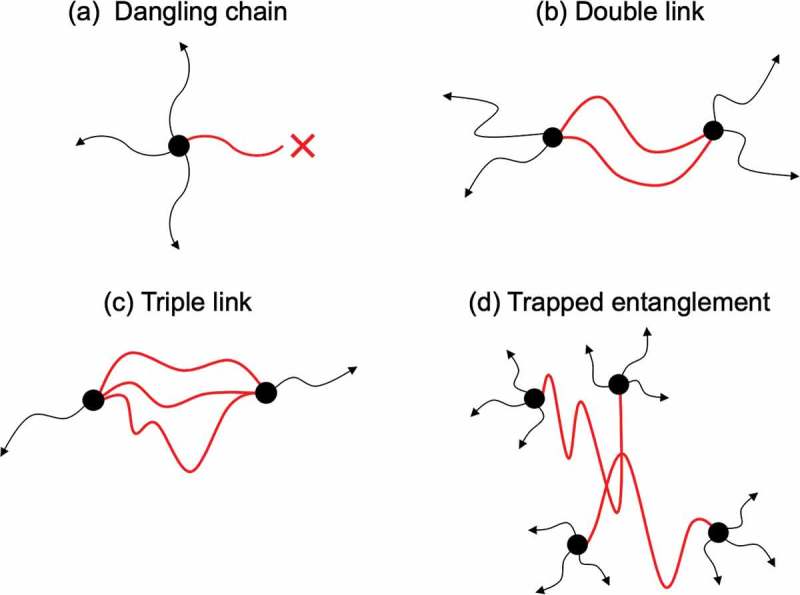
Microscopic heterogeneities in Tetra-PEG gel: (a) dangling chain, (b) double link, (c) triple link, and (d) trapped entanglement.

**Figure 3.**
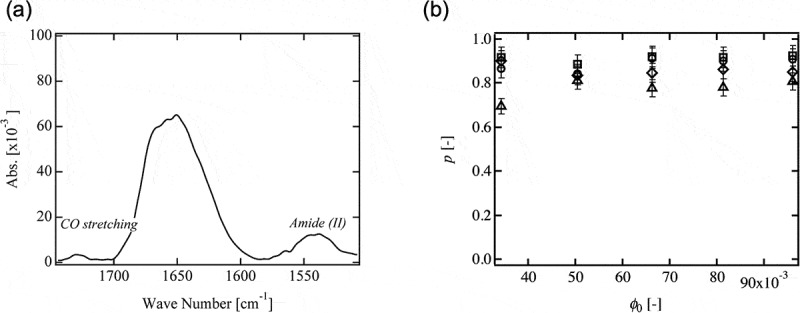
(a) Typical IR spectrum of dried Tetra-PEG gel (reproduced from Akagi et al. [16] with permission. Copyright 2011, ACS Publications). (b) The connectivity of the polymer network (*p*) of Tetra-PEG gel 5k (rhombuses), 10k (circles), 20k (squares) and 40k (triangles) (reproduced from Akagi et al. [19] with permission. Copyright 2013, ACS Publications).

Crosslinking freezes transient heterogeneity in a polymer solution at the gelation point and fixes as the permanent heterogeneity. Therefore, polymer gels are generally non-ergodic and heterogeneous materials [9]. Heterogeneity can be macroscopic and microscopic [8,9]. Microscopic heterogeneity is a kind of defects formed by a small number of chains, such as dangling chains, redundant connections (double and triple links) and trapped entanglements (Figure 2). Generally, these heterogeneities are hardly detected by any experiments; while the dangling chain can be directly estimated by spectroscopic experiment. To discuss the amount of dangling chain in Tetra-PEG gels, we measured the peak area of CO stretching in unreacted carboxyl group (*S*_CO_, 1725 cm^−1^) and that of reacted amide (Ⅱ) (*S*_amide (Ⅱ)_, 1545 cm^−1^) by Fourier transform infrared (FT-IR) absorption and estimated the fraction of connected end groups (*p*) by Equation (1) (Figure 3(a)) [16,19].

(1) $$p=\frac{S_{amide(\Pi )}}{S_{amide(\Pi )}+1.36\;S_{co}}$$

Here, the value of 1.36 is the ratio of the molar absorbance coefficient of CO stretching to amide (Ⅱ) estimated in advance.

We tuned the molecular weight (*M*_w_) and the initial polymer volume fraction (*ϕ*_0_) of the prepolymers and estimated *p* of Tetra-PEG gels (Figure 3(b)) [19]. The values of *p* were up to 0.9, and no appreciable dependences on *ϕ*_0_ and *M*_w_ were observed. Notably, *p* was almost constant below and above the overlapping concentration of the prepolymers (*ϕ*∗). This high connectivity of the polymer network suggests that the prepolymers are premixed homogeneously and react efficiently. Our previous research also confirmed the monodispersity and the high functionality of Tetra-PEG prepolymers [7]. These results indicate that Tetra-PEG gel satisfies the requirement (i); the length of all network strands should be identical.

**Figure 4.**
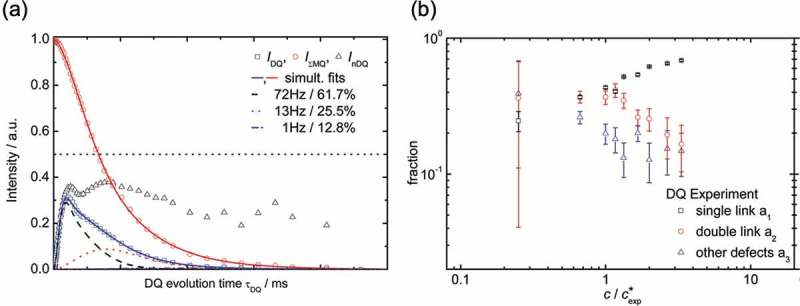
(a) ﻿Experimental data points for the double-quantum (DQ) buildup (*I*_DQ_, squares), the decay of the total multiple-quantum (MQ) magnetization (*I*_ΣMQ_, circles), and the normalized DQ intensity (*I*_nDQ_, triangles) of Tetra-PEG gel 10k (the polymer concentration is 120 g/L) (b) The relationship between fractions of ideal single link, double link and other higher order links and the concentration in Tetra-PEG gel (reproduced from Lange et al. [22] with permission. Copyright 2011, ACS Publications).

Although the direct observation of redundant connections had been considered difficult for a long time, it has been recently probed by ^1^H multiple-quantum nuclear magnetic resonance (NMR) [22]. Previous experiments showed only a broad relaxation in conventional polymer gels [23–25]; while Tetra-PEG gel showed some characteristic relaxation modes in the normalized double-quantum intensity (Figure 4(a)) [22]. These peaks are most likely assigned to characteristic structures with different relaxation times. From the concentration dependence of relaxation times, we identified each mode as an ideal single link, double link and other higher order links (Figure 4(b)). The fraction of an ideal single link was about 0.4 around the overlapping concentration and increased up to 0.7 with an increase in the concentration. The fraction of imperfect connections was about 0.3 in the higher concentration region. These results suggest that imperfect connections are suppressed in the higher concentration region, and Tetra-PEG gel nearly satisfies the requirement (iv); the functionality of crosslinking points should be constant throughout the entire network.

Macroscopic heterogeneity is the heterogeneous spatial distribution of polymer segments on the order of tens of nanometers to several micrometers, which is detected as an excess scattering in small angle X-ray scattering and small angle neutron scattering (SANS). The scattering curve (*I*(*q*)) of conventional polymer gels is represented by the superposition of the Ornstein–Zernike function (Equation (2)) [26], which corresponds to scattering from the homogeneous polymer solution, and the squared Lorentz or Gauss functions (Equation (3)), which are representative of the excess scattering [8,9,20,21].

(2) $$I\left(q\right)=\frac{{\left(\Delta \rho \right)}^{2}RT\phi^{2}}{N_{A}M_{os}}\frac{1}{1+\xi^{2}q^{2}}$$

(3) $$I\left(q\right)=\frac{{\left(\Delta \rho \right)}^{2}RT\phi^{2}}{N_{A}M_{os}}\frac{A_{hetero}}{{\left(1+\Xi^{2}q^{2}\right)}^{2}}$$

where (*Δρ*)^2^ is the square of the scattering length density difference between the polymer and solvent, *R* is the gas constant, *T* is the absolute temperature, *N*_A_ is Avogadro’s number, *ϕ* is the polymer volume fraction, *M*_OS_ is the longitudinal modulus, *ξ* is the correlation length of the network, *q* is the scattering vector, *Ξ* is the characteristic size of heterogeneities in the gel, and *A*_hetero_ is a constant representing the contribution of frozen heterogeneity.

**Figure 5.**
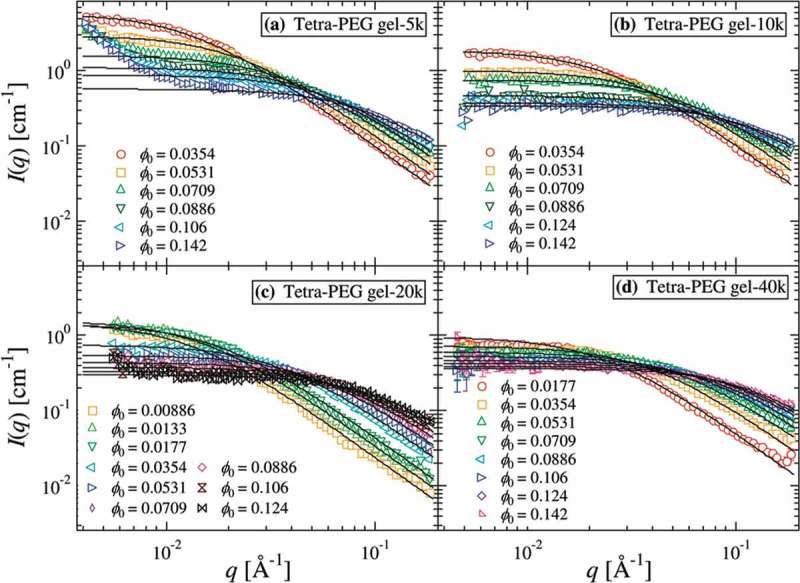
SANS intensity functions of Tetra-PEG gel (a) 5k, (b) 10k, (c) 20k, and (d) 40k. The solid lines denote the results of curve fitting with Ornstein-Zernike functions (reproduced from Matsunaga et al. [21] with permission. Copyright 2009, ACS Publications).

SANS results of Tetra-PEG gels are shown in Figure 5 [21]. In contrast to conventional polymer gels, the scattering curves of Tetra-PEG gels were reproduced only by the Ornstein–Zernike function in the region < 200 nm. Although the excess scattering was observed in the region > 200 nm, the *q*-dependence of the excess scattering of Tetra-PEG gels was much smaller than that of conventional polymer gels (Tetra-PEG gels: *I*(*q*) ~ *q* ^−2^, Conventional polymer gels: *I*(*q*) ~ *q* ^−4^). In addition, no appreciable excess scattering was observed even in the equilibrium-swollen state where spatial heterogeneity is emphasized. These results suggest that macroscopic heterogeneity is strongly suppressed in Tetra-PEG gels. In other words, Tetra-PEG gel satisfies the requirement (iii); the network should be homogeneous macroscopically as well as microscopically.

The above discussion demonstrates that spatial heterogeneity and microscopic heterogeneities are strongly suppressed in Tetra-PEG gels and that Tetra-PEG gel satisfies the requirements as the homogeneous network (i), (iii), and (iv). In the following section, we show that Tetra-PEG gel satisfies the requirement (v) from the aspect of fracture property. Therefore, Tetra-PEG gels satisfy the all requirements other than the requirement (ii) the Gaussian distribution of network strands, which is the inherent problem of polymer gels. As a result, we concluded that Tetra-PEG gel is suitable as a model network for the discussion of the structure-property relationship.

## 3. Gelation reaction

**Figure 6.**
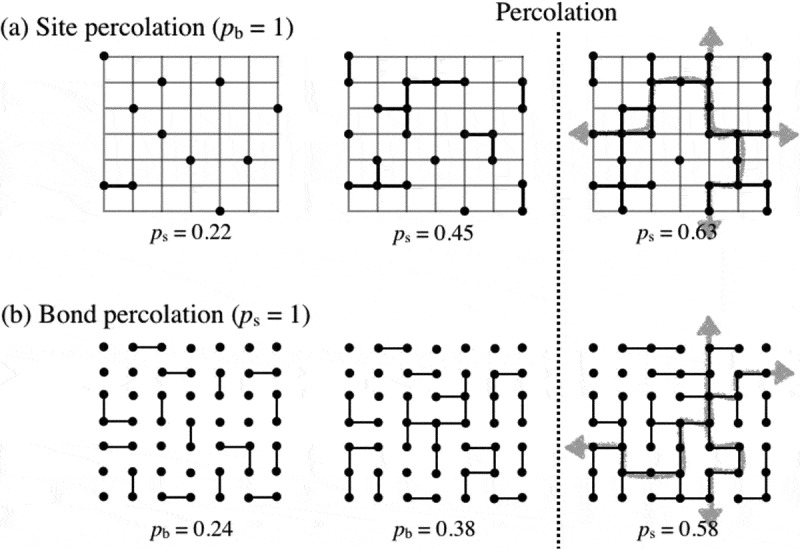
Prediction of gelation point by site percolation and bond percolation (reproduced from Sakai et al. [27] with permission. Copyright 2017, University of Tokyo Press).

Generally, the gelation process is explained in the context of the percolation [15,27]. Conventional percolation theory discusses the growth of clusters in a lattice space occupied by a site [28]. Percolation theory has two governing parameters: the fraction of occupied site and the fraction of connected site (Figure 6) [27,29]. There are analogies between the fraction of occupied site and the polymer concentration and between the fraction of connected site and the reaction conversion. The percolation theory with both fractions as variables is called the site-bond percolation model. We estimated the reaction conversion at the gelation point (*p*∗) by ^1^H NMR that measures the signals from the *β* carbon of the unreacted amino groups and that of the reacted amide bonds at various initial polymer volume fractions (*ϕ*_0_), and compared the results with the prediction of the site-bond percolation model [30].

**Figure 7.**
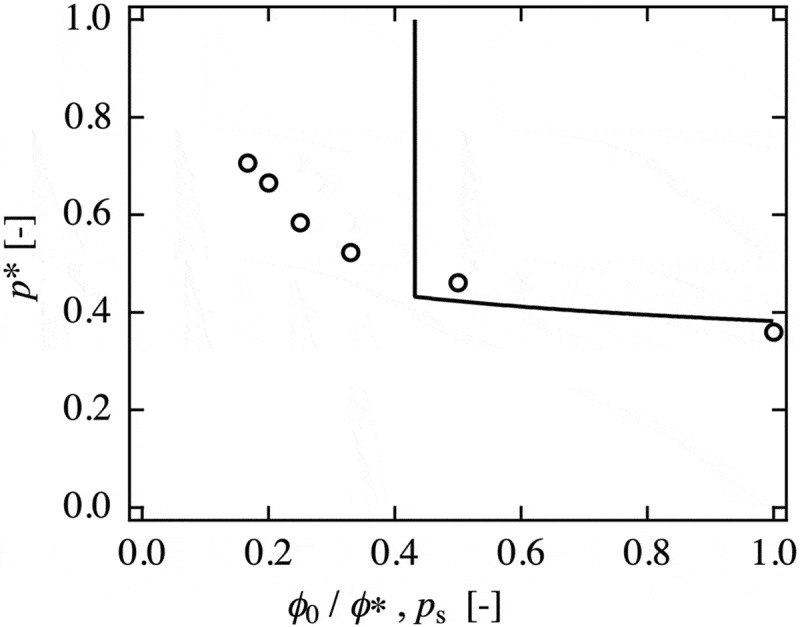
The relationship between the connectivity of the polymer network at the critical gelation point (*p*∗) and the normalized polymer volume fraction (*ϕ*_0_/*ϕ*∗) of Tetra-PEG gel. The solid line represents the prediction of the site-bond percolation model. (reproduced from Sakai et al. [30] with permission. Copyright 2016, The Society of Polymer Science, Japan (SPSJ)).

The relationship between *p*∗ and *ϕ*_0_ normalized by the overlapping concentration (*ϕ*_0_/*ϕ*∗) of Tetra-PEG gel is shown in Figure 7 [30]. The solid line in the figure represents the theoretical prediction of the site-bond percolation model. When we focused on the *ϕ*_0_/*ϕ*∗ ≈ 1 region, the experimental results were close to the theoretical prediction, suggesting the validity of the site-bond percolation model around *ϕ*∗. This correspondence indicates that the gels formed around the *ϕ*∗ have a homogeneous structure similar to that depicted in the model. However, a qualitative discrepancy was observed in the region much lower than *ϕ*∗. The theoretical prediction shows the discontinuous and clear concentration threshold; while the experimental *p*∗ increased with a decrease in *ϕ*_0_, and the gelation was observed even at *ϕ*∗/6. The discrepancy in this region may be because the prepolymer can diffuse during the gelation process, whereas the site does not diffuse in the lattice in the site-bond percolation model.

## 4. Elastic modulus

Elastic modulus (*G*), which is one of the most representative properties of solids, of a polymer gel is related to the number density of elastically effective chains (*ν*) and that of crosslinks (*μ*). There are three major models predicting elastic modulus: the affine (*G*_af_; Equation (4)) [31], phantom (*G*_ph_; Equation (5)) [32], and junction affine (*G*_ja_; Equation (6)) [33] network models.

(4) $$G_{af}=\nu k_{B}T$$

(5) $$G_{ph}=\left(\nu -\mu \right)k_{B}T$$

(6) $$G_{ja}=\left(\nu -h\mu \right)k_{B}T$$

where *k*_B_ is the Boltzmann constant, *T* is the absolute temperature, and *h* is the ﻿empirical parameter (0 ≤ *h* ≤ 1). The difference between these models is how to treat the fluctuation of crosslinking points. In the affine network model, it is assumed that crosslinking points are fixed and that the deformation of network strands is the same as the macroscopic deformation. Besides, the phantom network model assumes that the deformation of the network strands is suppressed by the fluctuation of crosslinking points. In the junction affine network model, which is an intermediate between these two models, the degree of the fluctuation is characterized by *h*. For a perfect tetra-functional polymer network, *G* predicted by the affine network model is twice as large as that by the phantom network model. Despite this significant difference, the validity or the applicable condition of these models are still unclear due to the heterogeneity in conventional gels. Furthermore, there is an experimental difficulty in the estimation of *ν* and *μ*.

An elastically effective chain is considered as the element of elasticity and defined as a chain whose both ends connect to crosslinks. Besides, a crosslink is defined as a junction point having three or more independent connections to the percolated gel network. The number densities of elastically effective chains and crosslinks (*ν* and *μ*) are calculated based on the tree approximation, which is one of the mean-field approaches [34,35]. The tree approximation is based on the recursive nature of the branching reaction and sets the following three assumptions.

1. All functional groups of the same type are equally reactive.
2. All groups react independently of one another.
3. No intramolecular reactions occur in finite species.

**Figure 8.**
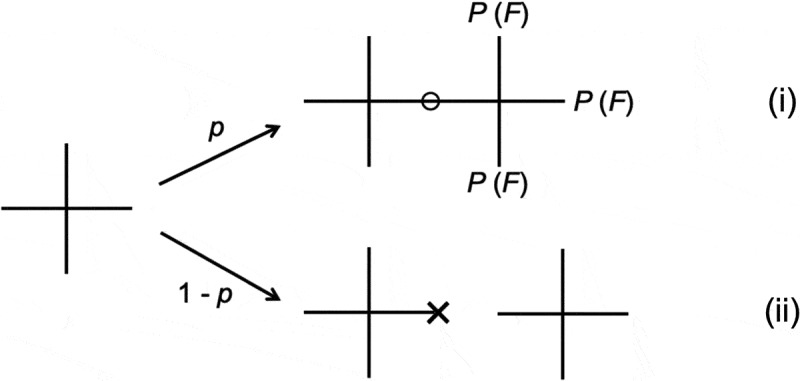
Calculation of the probability that an arm of a 4-armed polymer is not connected to an infinite network in the AA-type reaction (*P*(*F*)).

Based on these assumptions, we consider the AA-type reaction of 4-armed prepolymers, which is equivalent to the AB-type reaction in the stoichiometric condition. Here, AB-type reaction is a stepwise copolymerization of two types of monomers (A and B), and AA-type reaction is a stepwise homo-copolymerization of a monomer (A) [36]. We focus on one arm of a 4-armed prepolymer and consider the probability that the arm is not connected to the percolated network (*P*(*F*)). As shown in Figure 8, *P*(*F*) is the sum of the possibilities (i) that one arm connects to another 4-armed prepolymer and all remaining three arms of the connected 4-armed prepolymer do not connect to the percolated network and (ii) that the arm does not connect to another 4-armed prepolymer. Then, *P*(*F*) is written as

(7) $$P\left(F\right)=p\cdot P{\left(F\right)}^{3}+\left(1-p\right)$$

By solving Equation (7) for *P*(*F*), we obtain

(8) $$P\left(F\right)={\left(\frac{1}{p}-\frac{3}{4}\right)}^{\frac{1}{2}}-\frac{1}{2}$$

Using Equation (8), the probability that a 4-armed prepolymer becomes a 3-functional or 4-functional crosslink (*P*(*X*_3_) or *P*(*X*_4_)) is given as follows:

(9) $$P\left(X_{3}\right)=\left(\begin{array}{c} 4\\ 3 \end{array}\right)\cdot P\left(F\right)\cdot {\left[1-P\left(F\right)\right]}^{3}$$

(10) $$P\left(X_{4}\right)={\left[1-P\left(F\right)\right]}^{4}$$

Thus, *ν* and *μ* are written as

(11) $$\mu =c\cdot \left(P\left(X_{3}\right)+P\left(X_{4}\right)\right)$$

(12) $$\nu =c\cdot \left(\frac{3}{2}\cdot P\left(X_{3}\right)+2\cdot P\left(X_{4}\right)\right)$$

where *c* is the number density of 4-armed prepolymers. Based on Equations (4–12), one can estimate *G* from experimentally measured *p*.

**Figure 9.**
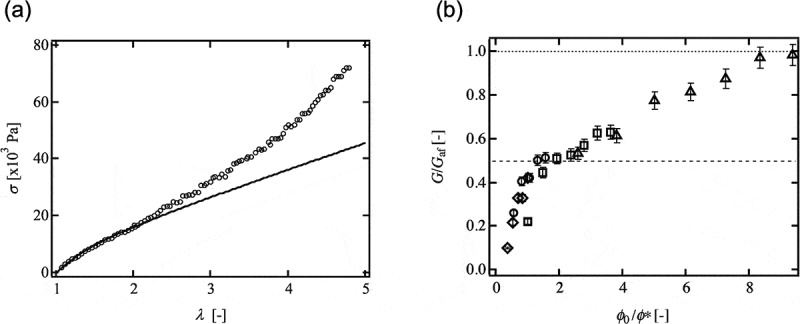
(a) Typical strass-strain relationship of Tetra-PEG gel and a fit by the neo-Hookean model (reproduced from Akagi et al. [16] with permission. Copyright 2011, ACS Publications). (b) The normalized elastic modulus (*G*/*G*_af_) as a function of the normalized polymer volume fraction (*ϕ*_0_/*ϕ*∗) (Tetra-PEG gel 5k, rhombuses; 10k, circles; 20k, squares; 40k, triangles; reproduced from Akagi et al. [19] with permission. Copyright 2013, ACS Publications).

To examine the validity of the abovementioned models, we measured *G* by tensile test and compared them with the theoretical predictions. The value of *G* was estimated from the initial slope of the stress-strain relationship (Figure 9(a)) [16]. For the low strain region, the stress-strain relationship of Tetra-PEG gel was well described by neo-Hookean model (*σ* = *G*(*λ* – *λ* ^−2^), *σ*: nominal stress, *λ*: nominal strain) [31]. This result suggests that *G* was precisely estimated by the tensile test. For the large strain region, the upward deviation due to the finite extensibility of the network strands was observed [37].

The relationship between *G*/*G*_af_ and *ϕ*_0_/*ϕ*∗ of Tetra-PEG gel is shown in Figure 9(b) [19]. Here, *ϕ*_0_ is the initial polymer volume fraction, and *ϕ*∗ is the overlapping polymer volume fraction of prepolymers. The affine and phantom network model predictions are the flat lines showing *G*/*G*_af_ = 1 (dotted line) and 0.5 (dashed line), respectively. All the data containing different molecular weight between crosslinks fall onto a single curve, suggesting *ϕ*_0_/*ϕ*∗ is one of the parameters governing the elasticity. In the range of *ϕ*∗ < *ϕ*_0_ < 3*ϕ*∗, *G* agreed well with *G*_ph_. Below *ϕ*∗, the values of *G* were smaller than that of *G*_ph_. On the other hand, above 3*ϕ*∗, *G*/*G*_af_ increased to approach 1 as *ϕ*_0_ increased. Generally, an increase of *G*/*G*_af_ with an increase in *ϕ*_0_ can be attributed to the introduction of trapped entanglements. However, the following discussion about fracture energy suggests that the effect of entanglements on the physical properties is negligible in Tetra-PEG gels [8,38]. Therefore, it seems that the result of Figure 9(b) suggests the transition of the model from the phantom to the affine network model with an increase in *ϕ*_0_.

The parameters tuned in the above discussion, the polymer concentration and the molecular weight between crosslinks, are expected to have a large influence on the system, resulting in the transition in the models. On the other hand, *p* is expected to have a minimal effect on the system, because one can change *p* without changing the materials consisting of the polymer gel. To investigate *G* in more detail, we investigated the relationship between *G* and *p* [39]. Experimentally, the time courses of *G* and *p* in the gelation process were measured by dynamic viscoelasticity measurement and ultraviolet-visible spectroscopic measurement, respectively. The relationship between *G* and *p* was obtained by using the time as a parametric variable.

**Figure 10.**
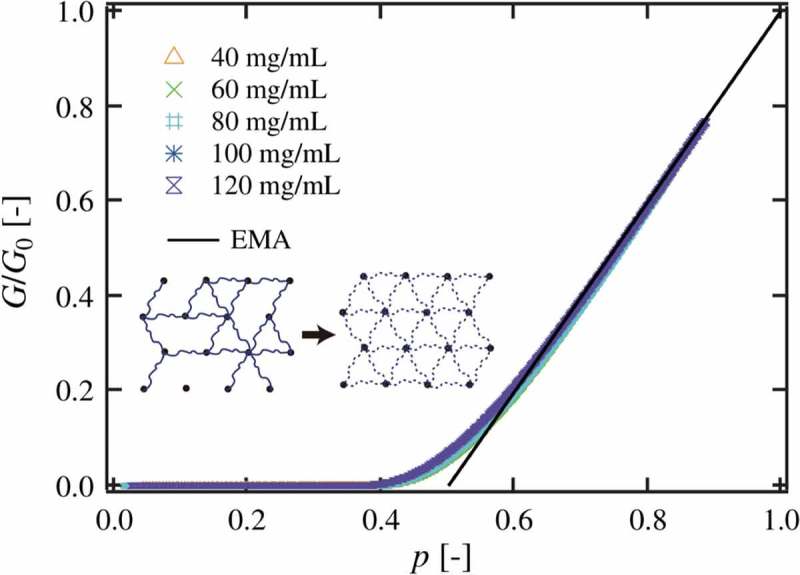
The connectivity of the polymer network (*p*) dependence of the normalized elastic modulus (*G*/*G*_0_) of Tetra-PEG gels. The solid line represents the prediction of effective medium approximation (EMA). (reproduced from Nishi et al. [39] with permission. Copyright 2017, American Physical Society).

In Figure 10, we plotted *G*/*G*_0_ against *p* [39]. Here, *G*_0_ is *G* at *p* = 1. All data with different *ϕ*_0_ fall onto a single curve, and well agreed with the prediction of effective medium approximation (EMA). EMA was originally developed to describe the conductivity of bond-disordered conductance network and was subsequently extended to describe the rubber elasticity of the Gaussian chain network [40,41]. As shown in the diagram of Figure 10, EMA converts a disordered network with elastic constant (*g*_0_) into an averaged ideal network with elastic constant (*g*_m_). Assuming that the average fluctuation of the crosslinks is zero under the balance of tension, *G*/*G*_0_ is described as the following equation in the range of large *p* (*p* > 0.75 in the case of the 4-branched network).

(13) $$G/G_{0}=\frac{g_{m}}{g_{0}}=\frac{p-\frac{2}{z}}{1-\frac{2}{z}}$$

where *z* is the number of branches of the network. It should be noted that the *G*/*G*_0_-*p* relationship in EMA agreed well with that in the phantom network model with the tree approximation. In other words, the *p*-dependence of *G*/*G*_0_ can be described by the phantom network model, and *G* is described as

(14) $$G=\left(\nu -\mu \right)g_{1}$$

The values of *g*_1_ were close to *k*_B_ *T* and were constant against *p* at *p* > 0.75. Besides, *g*_1_ increased with an increase in *ϕ*_0_, corresponding to the increase in *G*/*G*_af_ in Figure 9(b). This increase of *g*_1_ may be because the increase in polymer concentration suppresses the fluctuation of crosslinks and elastically effective chains.

## 5. Fracture energy

Fracture energy (*T*_0_) indicates the toughness of materials and is another important physical property of polymer gels. *T*_0_ is defined as the energy required to propagate a unit length of a crack from a crack tip [42]. The most famous model to predict *T*_0_ of elastic materials is Lake-Thomas model [43]. Lake-Thomas model describes *T*_0_ as the energy needed to break the chemical bonds per unit cross-section on the fracture surface as follows:

(15) $$T_{0}={\left(\frac{3}{8}\right)}^{\frac{1}{2}}\nu LNU$$

where *L* is the displacement length, *N* is the degree of polymerization of network strand, and *U* is the total bond energy of monomeric unit. In the original paper, the value of *L* is set to the length similar to an averaged strand length of the network (*R*_0_). Thus, using a constant *k*, Equation (15) is rephrased as follows:

(16) $$T_{0}={\left(\frac{3}{8}\right)}^{\frac{1}{2}}k\nu R_{0}NU$$

**Figure 11.**
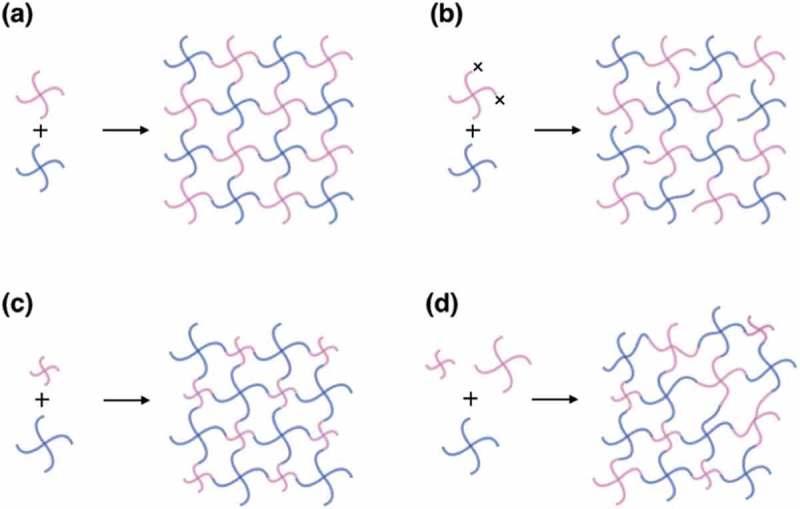
Schematic pictures of (a) conventional, (b) *p*-tuned, (c) hetero, and (d) bimodal Tetra-PEG gel (reproduced from Sakai et al. [44] with permission. Copyright 2014, The Royal Society of Chemistry).

To examine the validity of Lake-Thomas model, we performed a tearing test by using trouser-shaped Tetra-PEG gels [38,44]. We fabricated four kinds of Tetra-PEG gels: (a) conventional Tetra-PEG gel, (b) *p*-tuned Tetra-PEG gel, (c) hetero Tetra-PEG gel, and (d) bimodal Tetra-PEG gel (Figure 11). Notably, hetero Tetra-PEG gel is formed from A- and B-type prepolymers with different molecular weights, while *N* is monomodal. On the other hand, bimodal Tetra-PEG gel is formed from three kinds of prepolymers, including two A-type prepolymers with different molecular weights, resulting in bimodal distribution in *N*.

**Figure 12.**
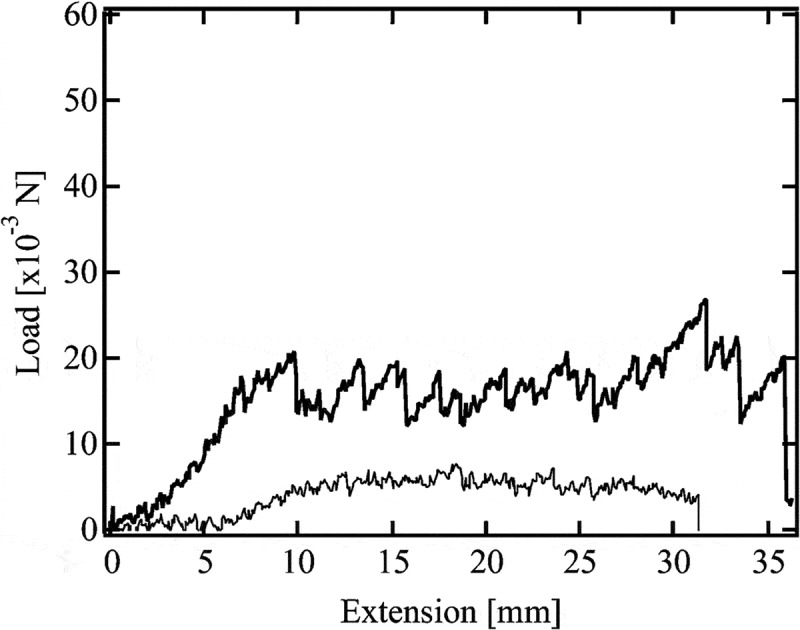
Typical tearing force-extension relationship of Tetra-PEG gel (reproduced from Sakai et al. [44] with permission. Copyright 2014, The Royal Society of Chemistry).

Figure 12 shows the typical tearing behavior of Tetra-PEG gel [44]. In the beginning, the load value monotonously increased with an extension, where crack did not propagate. After starting the crack propagation, the load fluctuated with an extension (stick-slip tearing). We estimated different values of *T*_0_ from the average of local maximum, the simple average and the average of local minimum values of the load (*F*) as,

(17) $$T_{0}=\frac{2F}{h}$$

where *h* is the thickness of the gel samples. The values of *T*_0_ estimated from the local maximum and average values of *F* were strongly influenced by the magnitude of stick-slip behavior, while *T*_0_ estimated from the local minimum values was negligibly affected by the magnitude of stick-slip behavior and systematically changed against the feed conditions. Although we realize the importance of the understanding of the stick-slip behavior, here, we focus on *T*_0_ estimated from the minimum values of *F*, which indicates the minimum energy required to propagate a crack.

**Figure 13.**
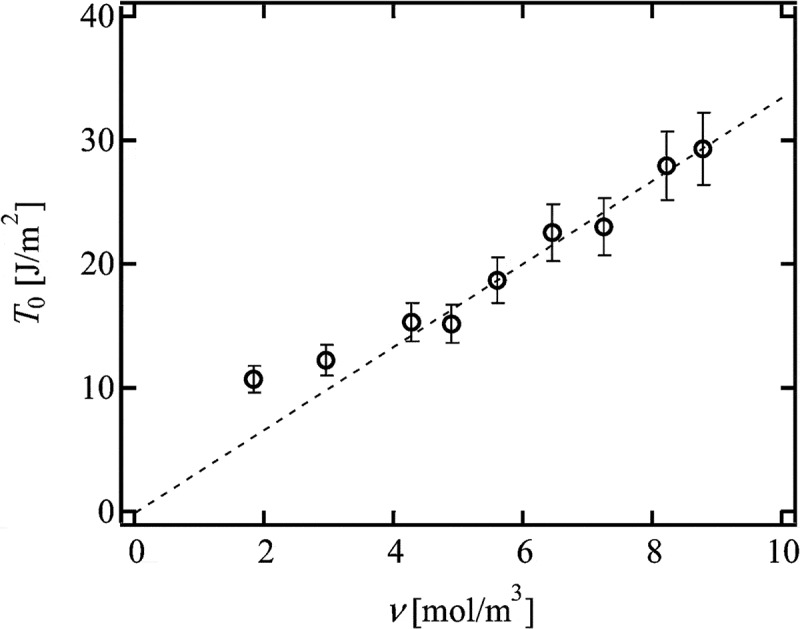
The relationship between *T*_0_ and *ν* in *p*-tuned Tetra-PEG gel (reproduced from Akagi et al. [38] with permission. Copyright 2013, AIP Publishing LLC).

The relationship between *T*_0_ and *ν* of *p*-tuned Tetra-PEG gel is shown in Figure 13 [38]. The dashed line represents the scaling prediction of Lake-Thomas model (*T*_0_ ~ *ν*). As shown in the figure, the experimental results obeyed Lake-Thomas model in the region *ν* > 4.0 (roughly corresponding to the region *p* > 0.7). This agreement indicates that *ν* calculated by Equation (12) is applicable to Lake-Thomas model and that the term *LNU* does not depend on *p* in the high *p* region. The discrepancy of the experimental results from Lake-Thomas model in the region *ν* < 4.0 is most probably due to the increase in *N* in low *p* region.

**Figure 14.**
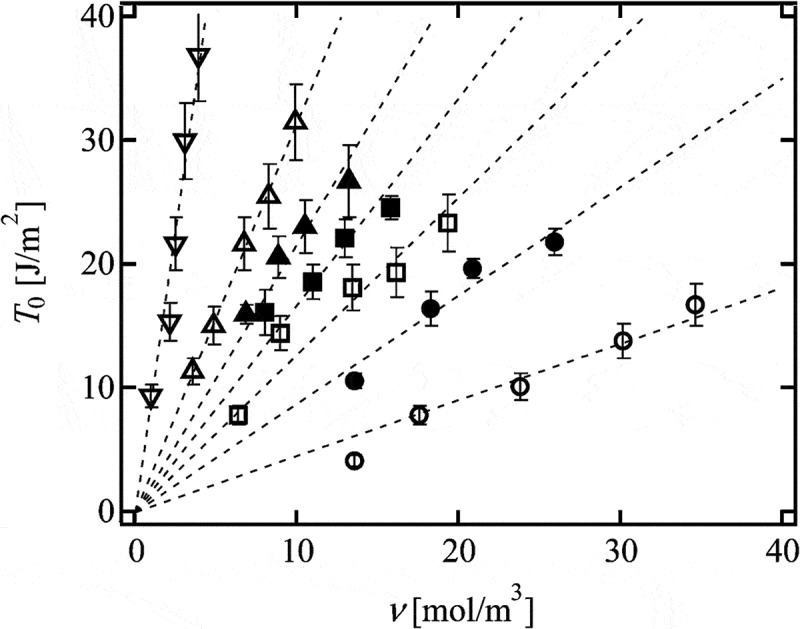
The relationship between *T*_0_ and *ν* in conventional and hetero Tetra-PEG gel. (conventional Tetra-PEG gel 5k, open circles; conventional Tetra-PEG gel 10k, open squares; conventional Tetra-PEG gel 20k, open triangles; conventional Tetra-PEG gel 40k, open inverted triangles; hetero Tetra-PEG gel 5k-10k, solid circles; hetero Tetra-PEG gel 5k-20k, solid squares; hetero Tetra-PEG gel 10k-20k, solid triangles; reproduced from Sakai et al. [44] with permission. Copyright 2014, The Royal Society of Chemistry.)

Figure 14 shows the values of *T*_0_ of conventional and hetero Tetra-PEG gel as a function of *ν* [44]. We observed the linear relationship between *T*_0_ and *ν* for each sample with an identical *N*, corresponding to the prediction of Lake-Thomas model (*T*_0_ ~ *ν*). Notably, we do not consider the existence of trapped entanglement, which is known to decrease *T*_0_ [45,46]. Thus, the agreement between the experimental results and the prediction of Lake-Thomas model suggests both the validity of the model and the absence of trapped entanglement in Tetra-PEG gels.

**Figure 15.**
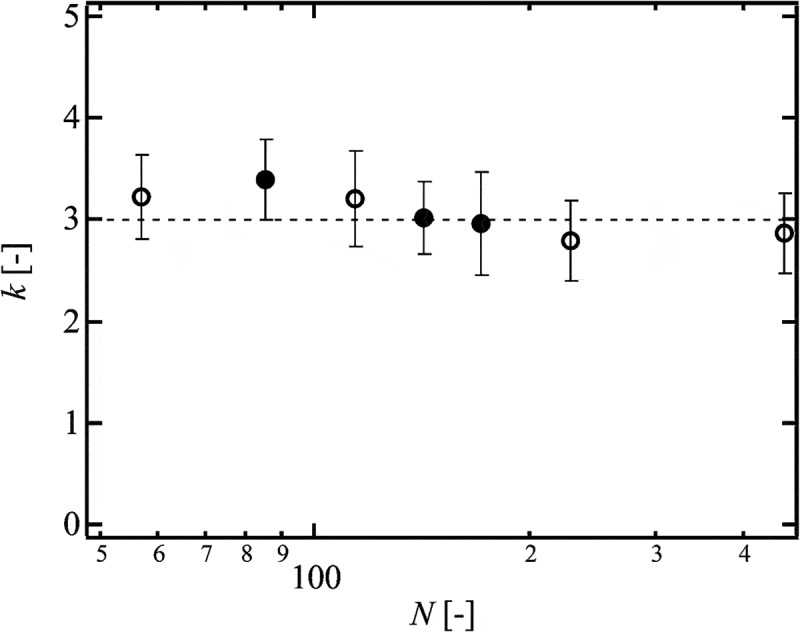
The relationship between *k* and *N* in conventional (open symbols) and hetero (solid symbols) Tetra-PEG gel (reproduced from Sakai et al. [44] with permission. Copyright 2014, The Royal Society of Chemistry).

Because the values of *R*_0_, *N, U* are estimated from the initial materials, we can estimate *k* from the slopes of the lines in Figure 14 based on Equation (16). Figure 15 shows the values of *k* of conventional and hetero Tetra-PEG gels as a function of *N* [44]. The values of *k* were almost constant and approximately 3 in this experimental region. This result indicates that the network strands within 3*R*_0_ from a crack tip are fully extended at the fracture, and the length is determined only by *N*, regardless of *p* and *ϕ*_0_. Although the values of *k* of bimodal Tetra-PEG gels were slightly smaller than those of conventional and hetero Tetra-PEG gel, the values were similar to each other [44]. This result suggests that the heterogeneous distribution in strand length does not significantly influence *T*_0_.

As discussed above, the initial polymer volume fraction (*ϕ*_0_), the degree of polymerization of a network strand (*N*), the connectivity of the polymer network (*p*), and the heterogeneous distribution in strand length did not influence the constant, *k*. From this robustness of *k*, it is expected that *k* does not change significantly even by changing the backbone structure of polymer gels.

## 6. Kinetics of swelling

**Figure 16.**
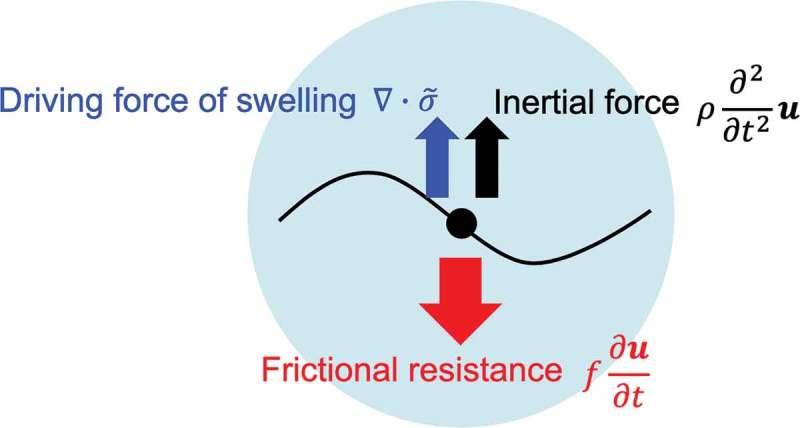
Schematic illustration of swelling equation.

When an as-prepared gel is soaked into a solvent, it conventionally swells [1,31]. The swelling should be managed especially for biomedical application of hydrogels [1,47,48]. The swelling of a gel is considered as the diffusion process of a polymer network to the outer solution. Therefore, the kinetics of swelling is described as the equation of motion for a small deformation of a unit cube in the network [49]. In general, the equation of motion of a unit cube moving in a fluid is given as,

(18) $$\rho \frac{\partial^{2}}{\partial t^{2}}u=\nabla \cdot \tilde{\sigma }-f\frac{\partial }{\partial t}u$$

where *ρ* is the density of a network, *t* is the time, ***u*** is the displacement vector, *σ* is the stress tensor, and *f* is the friction coefficient between polymer network and water. Here, the left side term, the first term on the right side, and the second term of the right side of the equation represent the inertial force, the force flowing into a unit cube (the driving force of swelling), and the hydrodynamic frictional resistance, respectively (Figure 16). Under spherical symmetry, Equation (18) gives

(19) $$\frac{\partial u}{\partial t}=\frac{K+\frac{4}{3}G}{f}\frac{\partial }{\partial r}\left\{\frac{1}{r^{2}}\left[\frac{\partial }{\partial r}\left(r^{2}u\right)\right]\right\}$$

where *K* is the bulk modulus, *G* is the shear modulus, and *r* is the displacement of a point. Here, *K* is the bulk modulus for non-poroelastic materials and relates to the equation of condition of polymer gels [20,50,51]. Because Equation (19) is similar to the diffusion equation, it is called the *swelling equation* [52], and the cooperative diffusion coefficient of a polymer gel (*D*_coop_) is defined as

(20) $$D_{coop}=\frac{K+\frac{4}{3}G}{f}$$

By applying the initial condition and the boundary condition to Equation (19) [53], the following solution is obtained.

(21) $$d_{n}=\frac{d_{\infty }-d\left(t\right)}{d_{\infty }-d_{0}}=\frac{6}{{\text{π}}^{2}}exp\left(-\frac{t}{\tau }\right)$$

where *d*(*t*) is the diameter of a gel at time *t, d*_0_ is the diameter in the initial state, *d*_∞_ is the diameter in the equilibrium swollen state, and *τ* is the longest characteristic time of swelling. The values of *d*_∞_ and *τ* are related to *D*_coop_ as

(22) $$D_{coop}=\frac{d_{\infty }^{2}}{{\text{π}}^{2}\tau }$$

**Figure 17.**
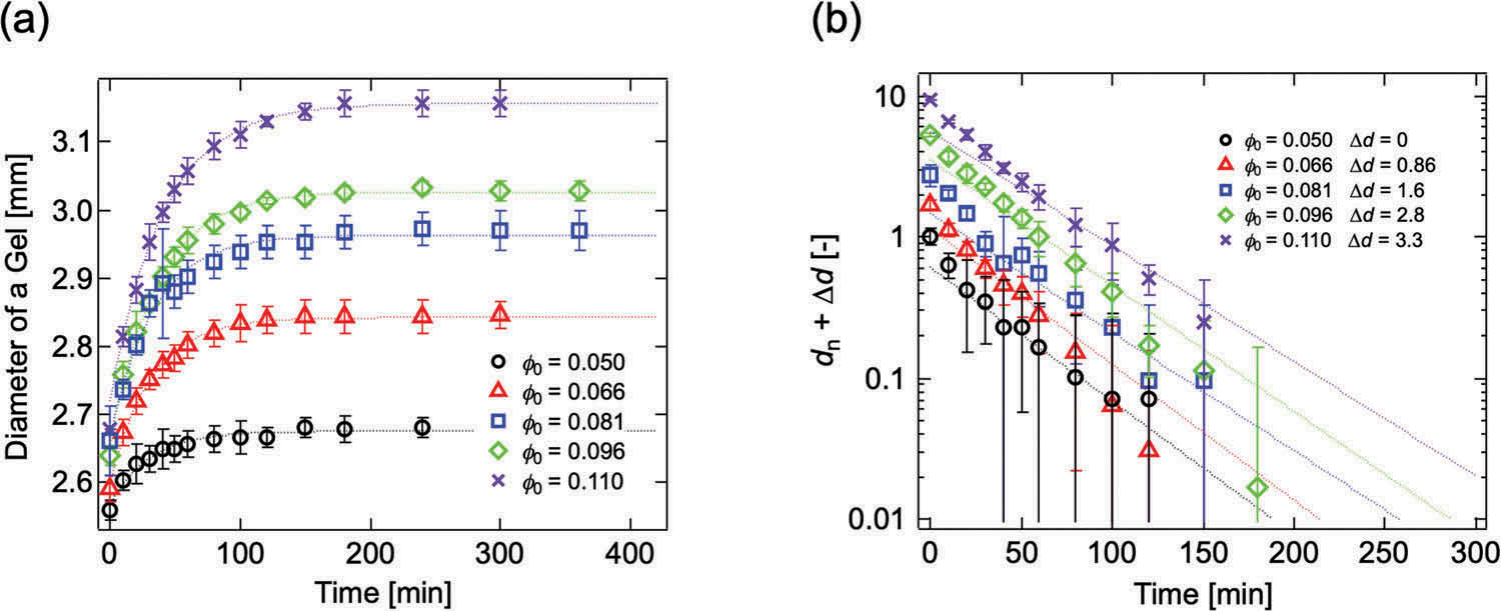
(a) The typical swelling curves of Tetra-PEG gel 10k, (b) The typical time courses of the normalized diameter of a gel (*d*_n_) during the swelling experiments for Tetra-PEG gel 10k (reproduced from Fujiyabu et al. [50] with permission. Copyright 2018, The Royal Society of Chemistry).

To examine the validity of the *swelling equation*, we conducted a swelling experiment using Tetra-PEG gels with different network structure parameters [50]. The swelling behavior of Tetra-PEG gels is shown in Figure 17. The experimental results were well reproduced by Equation (21) (dotted lines), suggesting the validity of the *swelling equation*.

Then, we focused on the cooperative diffusion coefficient (*D*_coop_). The value of *D*_coop_ is estimated not only by swelling experiment (*D*_sw_, Equation (22)) but also by the definition of *D*_coop_ (*D*_w_, Equation (20)) and by dynamic light scattering (DLS) experiment (*D*_DLS_). In previous studies, these three *D*_coop_’s were considered to be identical to each other (*D*_coop_ = *D*_w_ = *D*_sw_ = *D*_DLS_) [49,53]. To investigate the identicality of three *D*_coop_’s, we estimated and compared them [50]. Here, we calculated *D*_w_ by Equation (20) using the values of *K, G* and *f* estimated from macroscopic mechanical experiments (*K*: osmotic pressure measurement, *G*: dynamic viscoelastic measurement, *f*: water permeation measurement), *D*_sw_ by Equation (22) based on the result of Figure 17, and *D*_DLS_ by DLS based on the time-correlation function [50,54,55].

**Figure 18.**
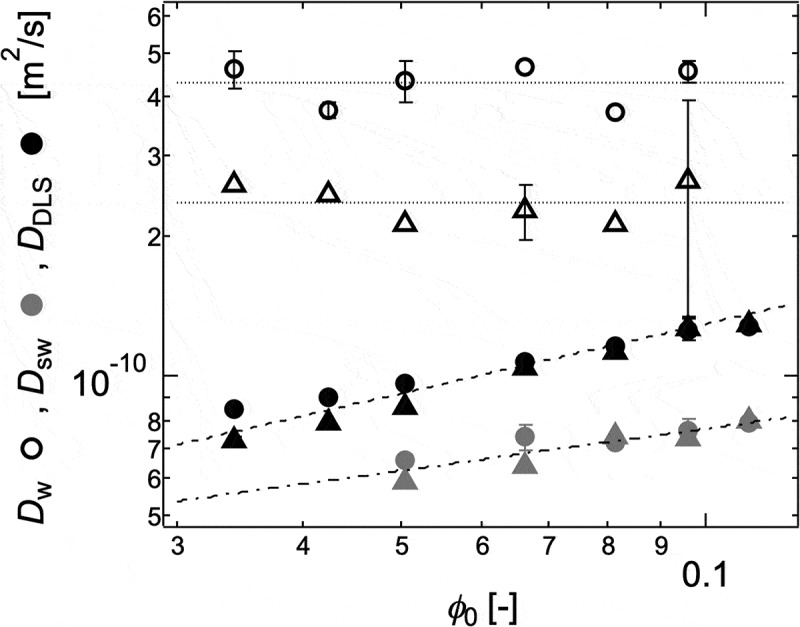
The *ϕ*_0_-dependences of *D*_w_ (white symbols), *D*_DLS_ (black symbols) and *D*_sw_ (gray symbols) of Tetra-PEG gel 10k (circles) and 20k (squares) (reproduced from Fujiyabu et al. [50] with permission. Copyright 2018, The Royal Society of Chemistry).

In Figure 18, *D*_w_, *D*_sw_, and *D*_DLS_ of Tetra-PEG gels are plotted against the initial polymer volume fraction (*ϕ*_0_) [50]. Three diffusion coefficients were divided into two types: *M*_w_-dependent *D*_w_ and *ϕ*_0_-dependent *D*_sw_ and *D*_DLS_. Theoretically, the cooperative diffusion of a polymer network is considered to be the diffusion of blobs (*D*_coop_ ~ *ξ* ^−1^; *ξ*: blob size) [15,56]. Because the relationship between *ξ* and *ϕ*_0_ in the semi-dilute solution of a good solvent is written as *ξ* ~ *ϕ*_0_^−0.75^, conceptually *D*_coop_ depends only on *ϕ*_0_. Because the *ϕ*_0_-dependence and *M*_w_-independence of *D*_sw_ and *D*_DLS_ roughly agreed with the concept of *D*_coop_, they can be considered as *D*_coop_ in the *swelling equation*. On the other hand, the behavior of *D*_w_ was completely different from those of *D*_sw_ and *D*_DLS_. This discrepancy suggests the inapplicability of *f* estimated from water permeation experiment in the calculation of *D*_coop_ (Equation (20)). In other words, *f* estimated from water permeation describes the motion of water molecules [54], while *D*_coop_ describes the motion of a polymer network. These results suggest that the swelling of a gel is governed by the diffusion of a polymer network to the outer solution, but not by those of water molecules, as originally suggested by T. Tanaka [49,53].

## 7. Conclusions

In this review, we showed the structure-property relationship of a model polymer network system containing a solvent (Tetra-PEG gel). The homogeneity of Tetra-PEG gel has been confirmed by SANS, IR absorption and ^1^H multiple-quantum NMR. The AB-type reaction of tetra-functional prepolymers in the semi-dilute region strongly contributes to the structural homogeneity of Tetra-PEG gel. In addition, the optimization of reaction rate realizes the reaction-limited gelation reaction, resulting in a homogeneous mixing of prepolymers. The gelation process of Tetra-PEG gel was roughly understood in the context of the percolation model.

Elastic modulus, which characterizes the small deformation, was described by the equation similar to the phantom network model. On the other hand, there are still unknowns about the concentration dependence of elastic modulus. We are tackling this problem by investigating the effects of solvent quality and temperature on elastic modulus.

Fracture property, which characterizes the large deformation, was well described by the Lake-Thomas model with reasonable parameters predicted by the chemical crosslinking. This may be because the effect of the initial conformation of polymer chains is negligible in the large deformation. In the near future, we will investigate the crack propagation behavior in detail.

Kinetics of swelling is a key property of polymer networks containing solvents. Our experimental results agree well with the conventional theory proposed by T. Tanaka [49,53]. These results strongly support the concept that the swelling of a gel is the diffusion process of a polymer network to the outer solution. In the future, we will investigate the relationships between the cooperative diffusion of a polymer network and the diffusion of solvent molecules and between static blob estimated by neutron scattering and dynamic blob estimated by cooperative diffusion coefficient for the further understanding of dynamics in polymer gels.

The presence of a solvent reveals a different aspect of polymer networks, which cannot be observed without a solvent. It simply provides the additional parameter, polymer concentration. In addition, the suppression of trapped entanglements enables the independent control over the number of crosslinks and the molecular weight between crosslinks. Notably, the conformation of polymer chains is not ideal in polymer gels, and the non-ideality should influence the physical properties. We will tackle the non-ideality problem in the near future. We hope these results will help understanding polymer networks, which is one of the indispensable materials in our daily life.
